# Rapid Recurrence of Giant Multilocular Prostatic Cystadenoma after Laparoscopic Excision for Primary Case: A Case Report

**DOI:** 10.3390/medicina57090870

**Published:** 2021-08-25

**Authors:** Tae-Soo Choi, Dong-Gi Lee, Koo-Han Yoo, Gyeong-Eun Min

**Affiliations:** Department of Urology, College of Medicine, Kyung Hee University, Seoul 05278, Korea; taesoochoi85@hanmail.net (T.-S.C.); urology@khu.ac.kr (D.-G.L.); koohanyoo@khu.ac.kr (K.-H.Y.)

**Keywords:** prostate, cystadenoma, laparoscopy, surgery

## Abstract

Giant multilocular prostatic cystadenoma is a rare benign tumor of the prostate gland that presents as a large retroperitoneal pelvic mass. The mass is usually located between the urinary bladder and rectum, and results in obstructive voiding symptoms and a change in bowel habits. Complete surgical excision is the treatment of choice. We present a case of rapid recurrent giant multilocular prostatic cystadenoma after laparoscopic excision for primary case. A previously healthy 54-year-old man presented with acute urinary retention. Prostate MRI showed a large cystic mass approximately 13 cm in size, multiple septa and lobulation in the prostate, and no visible solid lesions. Laparoscopic marsupialization of giant multilocular prostatic cystadenoma cysts was performed. One year later, the patient presented with local recurrence. Repeated laparoscopic complete resection was performed without any complications and further recurrence. Giant multilocular prostatic cystadenoma has the risk of recurrence in case of incomplete resection. Surgical treatment should be performed with the goal of complete removal following the same principles as cancer surgery.

## 1. Introduction

Giant multilocular prostatic cystadenoma is a rare benign tumor of the prostate gland that presents as a large retroperitoneal pelvic mass [1,2]. The mass is usually located between the urinary bladder and the rectum, and results in obstructive voiding symptoms and a change in bowel habits [1,3]. Magnetic resonance image (MRI) is used to characterize the giant multilocular prostatic cystadenoma, and findings demonstrate a large retroperitoneal mass with multiple and variable-sized cysts compressing adjacent organs, especially the bladder and rectum [4]. Here, we present a case of rapid recurrence of giant multilocular prostatic cystadenoma after laparoscopic excision of primary case.

## 2. Case Report

**Figure 1:**
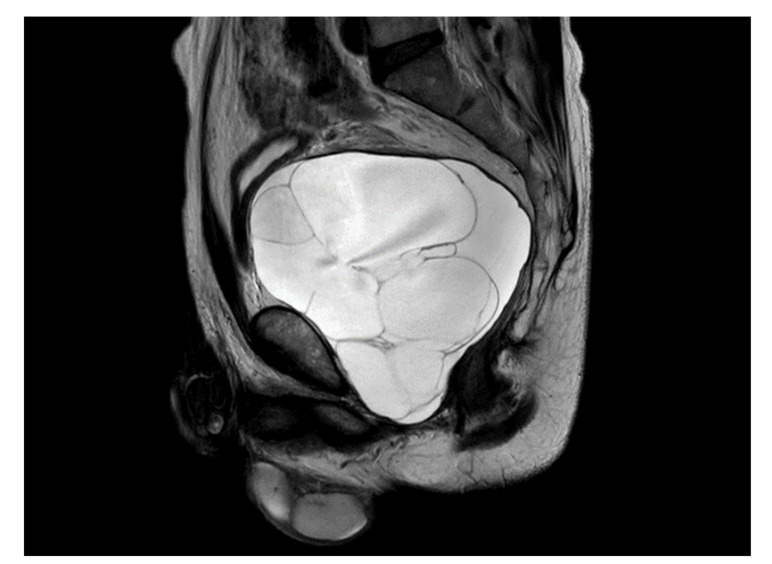
Prostate MRI showed a large cystic mass approximately 13 cm in size, multiple septa and lobulating contours in the prostate, and no visible solid lesions.

A previously healthy 54-year-old man presented with acute urinary retention. The patient also presented with weak stream, tenesmus, hesitancy, and intermittency. On digital rectal examination, the enlarged prostate was palpated softly, and no nodules were detected. His PSA level was 0.469 ng/mL. Prostate MRI showed a large cystic mass approximately 13 cm in size, multiple septa and lobulation in the prostate, and no visible solid lesions (Figure 1). The urinary bladder and rectum were displaced as a result of the enlarged cystic prostatic mass.

**Figure 2:**
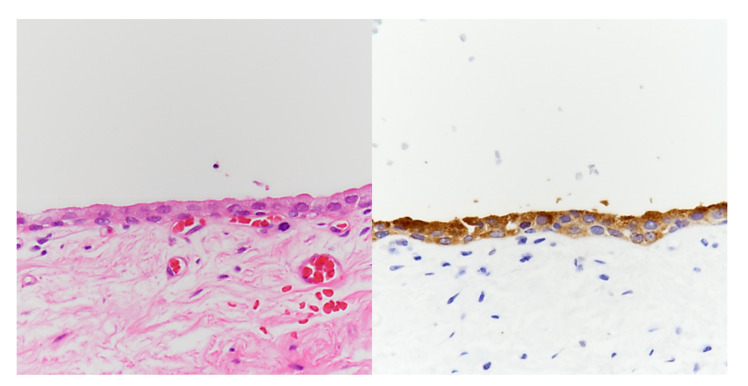
Histologically, the giant multilocular prostatic cystadenoma is lined by flat to cuboidal epithelium set in the hypocellular fibrous stroma. No cytologic atypia is identified (left, H-E, ×400). The epithelial lining displays PSA immunopositivity, confirming its prostatic origin (right, PSA, ×400).

After confirming the benign nature of the lesions through the transrectal needle biopsy, laparoscopic surgery was performed through four trocars (a 12-mm port for the camera, two 11-mm working ports, and one 5-mm working port) used in the same configuration as the laparoscopic prostatectomy procedure. The patient had fear of urinary incontinence and erectile dysfunction following the radical surgery; therefore, he decided to receive the marsupialization of cystic mass. After opening the peritoneum overlying the cystic mass, the lateral part of the tumor was dissected first, followed by the medial aspect of the tumor. The large cystic mass was isolated from the urinary bladder and rectum, and more than 300 cc of clear fluid was aspirated from the cystic mass. Following collapse of the cystic mass, serial marsupialization of multiple cysts was performed with blunt and sharp dissection. Gross pathologic examination revealed a 12.0 × 9.0 × 1.0 cm-sized mass with multiple cysts without solid portion. Microscopically, the cystic wall was lined with flat to cuboidal prostatic epithelia and was immunoreactive for PSA (Figure 2). Finally, the lesion was diagnosed as a giant multilocular prostatic cystadenoma.

**Figure 3:**
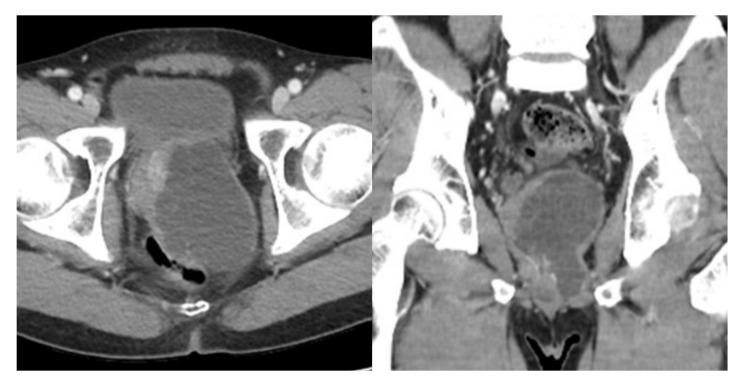
Abdominal CT showed an 8.0 × 6.5 × 9.0 cm-sized, lobulated cystic lesion with multiple thin septa in the left prostate without solid component.

There were no complications postoperatively, and the patient was discharged on postoperative day 5. At the 12-month follow-up, computed tomography (CT) revealed the recurred mass in the previous surgical field, about 8.0 × 6.5 × 9.0 cm-sized, lobulated cystic lesion with multiple thin septa in the left prostate without solid component (Figure 3). Repeated laparoscopic complete resection of the mass was performed by dissecting the base of the cyst from the normal prostate tissue. The patient fully recovered after surgery without erectile dysfunction, urinary incontinence, and further recurrence.

## 3. Discussion

Giant multilocular prostatic cystadenoma is a rare benign tumor of the prostate gland. Since Maluf et al. reported the first case in 1991, fewer than 30 cases have been reported [1,2,3]. Prostatic cystadenoma is usually characterized by large multilocular cysts that are located between the urinary bladder and the rectum. The clinical presentation includes obstructive voiding symptoms, such as weak stream, intermittency, straining, vesical tenesmus, and urinary retention. Constipation and decreased stool caliber can also occur as a result of compression of the rectum [1,2,5,6,7,8,9,10,11,12,13].

CT and MRI of giant multilocular prostatic cystadenomas demonstrate the cystic nature of the lesions, which are of various sizes, with multiple septa compressing the bladder and rectum, and without invasive large solid enhancing component [4,10,13]. With the development of the MRI technique, it is now possible to clarify the exact boundaries of lesions and whether or not they invade surrounding organs, which is helpful in determining the treatment method and pre-surgical planning [4].

Differential diagnoses for cystic retroperitoneal mass include simple prostatic cyst, phyllodes variant of benign prostatic hyperplasia, prostatic sarcoma, prostatic leiomyoma, echinococcal cysts of the prostate, Mullerian duct cysts, cystic dilatation of the utricle, diverticulum of the ejaculatory duct or ampulla of the vas deferens, seminal vesicle cysts, pelvic mesothelioma, multilocular peritoneal inclusion cyst, cystic teratoma, lymphangioma, and prostatic cystic carcinoma [2].

Microscopically, giant multilocular prostatic cystadenoma is characterized by typical prostatic glands and cysts lined with double layers of columnar and cuboid cells with basally located nuclei and pale cytoplasm [13]. The epithelial cells usually show positive PSA staining, which can be helpful in distinguishing other lesions [2,13].

Surgical managements for giant multilocular prostatic cystadenoma have been reported, including transurethral resection or enucleation of the prostate, laparoscopic cyst resection, partial or complete tumor resection, and even radical prostatectomy or pelvic exenteration [14]. Giant multilocular prostatic cystadenoma is pathologically benign, but might be clinically malignant. Following transurethral resection of the giant multilocular prostatic cystadenoma and incomplete tumor resection, recurrence has been reported in a small number of cases [14,15]. Remnant tissue of the giant multilocular prostatic cystadenoma could be the cause of recurrence. Complete surgical excision is the treatment of choice for giant multilocular prostatic cystadenoma to reduce the risk of recurrence [3]. Most of the cases reported so far have been treated through laparotomy rather than a minimal invasive approach. Although laparoscopic resection has been reported for recurrent cases, there have been no cases in which the primary and recurrent case of same patient was treated with a minimally invasive approach [16]. Even in the case of recurrence after laparoscopic surgery, reoperation is feasible, because there is little adhesion in the abdominal cavity. In the treatment of prostate cancer, it has been demonstrated that minimally invasive treatment using laparoscopic or robotic surgery compared to open surgery have many advantages in terms of less pain, quick recovery, and good functional outcomes with regards to preservation of sexual function and urinary continence [17]. In cases of giant multilocular prostatic cystadenoma, it is better to apply minimally invasive treatment if possible in order to reduce the risk of sexual dysfunction or urinary incontinence due to the anatomical location of the tumor. With the development of the MRI technique, it is now possible to clarify the exact boundaries of lesions and whether or not they invade surrounding organs, which is helpful in determining the treatment method and presurgical planning [4]. Since the cystadenoma does not invade surrounding tissues, it can be completely removed, even with minimal invasive treatment [2].

In conclusion, giant multilocular prostatic cystadenoma has a risk of recurrence in case of incomplete resection. Surgical treatment should be performed with the goal of complete removal, following the same principles as cancer surgery.

## Data Availability

The data reported in this paper are available from the medical history of the patient.

## References

[1] Watanabe J., Konishi T., Takeuchi H., Tomoyoshi T. (1990). A case of giant prostatic cystadenoma. Hinyokika Kiyo.

[2] Maluf H.M., King M.E., DeLuca F.R., Navarro J., Talerman A., Young R.H. (1991). Giant multilocular prostatic cystadenoma: A distinctive lesion of the retroperitoneum in men. A report of two cases. Am. J. Surg. Pathol..

[3] Nakamura Y., Shida D., Shibayama T., Yoshida A., Matsui Y., Shinoda Y., Iwata S., Kanemitsu Y. (2019). Giant multilocular prostatic cystadenoma. World J. Surg. Oncol..

[4] Baad M., Ericson K., Yassan L., Oto A., Eggener S., Nottingham C.U., Richards K.A., Thomas S. (2015). Giant Multilocular Cystadenoma of the Prostate. Radiographics.

[5] Levy D.A., Gogate P.A., Hampel N. (1993). Giant multilocular prostatic cystadenoma: A rare clinical entity and review of the literature. J. Urol..

[6] Lim D.J., Hayden R.T., Murad T., Nemcek A.A., Dalton D.P. (1993). Multilocular prostatic cystadenoma presenting as a large complex pelvic cystic mass. J. Urol..

[7] Kirsch A.J., Newhouse J., Hibshoosh H., O’Toole K., Ritter J., Benson M.C. (1996). Giant multilocular cystadenoma of the prostate. Urology.

[8] Seong B.M., Cheon J., Lee J.G., Kim J.J., Chae Y.S. (1998). A case of multilocular prostatic cystadenoma. J. Korean Med. Sci..

[9] Matsumoto K., Egawa S., Iwabuchi K., Baba S. (2002). Prostatic cystadenoma presenting as a large multilocular mass. Int. J. Urol..

[10] Rusch D., Moinzadeh A., Hamawy K., Larsen C. (2002). Giant multilocular cystadenoma of the prostate. AJR Am. J. Roentgenol..

[11] Allen E.A., Brinker D.A., Coppola D., Diaz J.I., Epstein J.I. (2003). Multilocular prostatic cystadenoma with high-grade prostatic intraepithelial neoplasia. Urology.

[12] Datta M.W., Hosenpud J., Osipov V., Young R.H. (2003). Giant multilocular cystadenoma of the prostate responsive to GnRH antagonists. Urology.

[13] Olgun D.C., Onal B., Mihmanli I., Kantarci F., Durak H., Demir H., Cetinel B. (2012). Giant multilocular cystadenoma of the prostate: A rare cause of huge cystic pelvic mass. Korean J. Urol..

[14] Fan L.W., Chang Y.H., Shao I.H., Wu K.F., Pang S.T. (2020). Robotic surgery in giant multilocular cystadenoma of the prostate: A rare case report. World J. Clin. Cases.

[15] Yang W.B., Zhang X.W., Yang J., Li Q., Xu T., Bai W.J. (2018). Transurethral resection of prostate treatment for recurrence of a multilocular prostatic cystadenoma: A case report. J. Peking Univ. Health Sci..

[16] El Rahman D.A., Zago T., Verduci G., Baroni G., Berardinelli M.L., Pea U., Morandi E., Castoldi M. (2016). Transperitoneal laparoscopic treatment for recurrence of a giant multilocular prostatic cystadenoma: A case report and review of the literature. Arch. Ital. Di Urol. Androl..

[17] Seo H.J., Lee N.R., Son S.K., Kim D.K., Rha K.H., Lee S.H. (2016). Comparison of Robot-Assisted Radical Prostatectomy and Open Radical Prostatectomy Outcomes: A Systematic Review and Meta-Analysis. Yonsei Med. J..

